# Nightshift Work and Nighttime Eating Are Associated With Higher Insulin and Leptin Levels in Hospital Nurses

**DOI:** 10.3389/fendo.2022.876752

**Published:** 2022-05-09

**Authors:** Hylton E. Molzof, Courtney M. Peterson, S. Justin Thomas, Gabrielle F. Gloston, Russell L. Johnson, Karen L. Gamble

**Affiliations:** ^1^Department of Psychiatry and Behavioral Sciences, Division of Sleep Medicine, Stanford University, Stanford, CA, United States; ^2^Department of Nutrition Sciences, University of Alabama at Birmingham, Birmingham, AL, United States; ^3^Department of Psychiatry and Behavioral Neurobiology, Heersink School of Medicine, University of Alabama at Birmingham, Birmingham, AL, United States

**Keywords:** circadian misalignment, meal timing, insulin, Leptin, shiftwork

## Abstract

**Background:**

Circadian misalignment between behaviors such as feeding and endogenous circadian rhythms, particularly in the context of shiftwork, is associated with poorer cardiometabolic health. We examined whether insulin and leptin levels differ between dayshift versus nightshift nurses, as well as explored whether the timing of food intake modulates these effects in nightshift workers.

**Methods:**

Female nurses (N=18; 8 dayshift and 10 nightshift) completed daily diet records for 8 consecutive days. The nurses then completed a 24-h inpatient stay, during which blood specimens were collected every 3 h (beginning at 09:00) and meals were consumed at regular 3-h intervals (09:00, 12:00, 15:00, and 18:00). Specimens were analyzed for insulin and leptin levels, and generalized additive models were used to examine differences in mean insulin and leptin levels.

**Results:**

Mean insulin and leptin levels were higher in nightshift nurses by 11.6 ± 3.8 mU/L (p=0.003) and 7.4 ± 3.4 ng/ml (p=0.03), respectively, compared to dayshift nurses. In an exploratory subgroup analysis of nightshift nurses, predominately eating at night (21:00 – 06:00) was associated with significantly higher insulin and leptin levels than consuming most calories during the daytime (06:00 – 21:00).

**Conclusions:**

In our study of hospital nurses, working the nightshift was associated with higher insulin and leptin levels, and these effects were driven by eating predominately at night. We conclude that although nightshift work may raise insulin and leptin levels, eating during the daytime may attenuate some of the negative effects of nightshift work on metabolic health.

## Introduction

Metabolism is modulated by the endogenous circadian system and exogenous environmental and behavioral factors (1). Several metabolic processes—including insulin sensitivity, insulin secretion, postprandial insulin levels, and leptin—exhibit rhythms across the 24-h day (2, 3). Some of these rhythms, such as in insulin secretion and sensitivity, are driven by the circadian system, whereas other metabolic rhythms such as leptin appear to be driven by external factors, including the timing of food intake (1, 2, 4, 5). In particular, insulin and leptin are key metabolic hormones that play an important role in glucose homeostasis and body weight through regulation of glucose metabolism, energy expenditure, and food intake (6). As such, there is interplay between the circadian system, behavioral factors (e.g., meal timing), and metabolic health.

Conversely, disruption of these circadian rhythms has been implicated in the development of cardiovascular and metabolic diseases (7–10). In studies that simulate shiftwork over short periods of time, light exposure in the evening/nighttime or during daytime sleep increases insulin levels (11–14). Similarly, acute circadian misalignment induced *via* a 28-h day Forced Desynchrony Protocol disrupts glucose, insulin, and leptin rhythms, resulting in higher glucose and insulin levels and lower leptin levels (5, 15). Interestingly, some of these effects may be mediated by changes in the timing of food intake, which has also been shown to affect key metabolic rhythms, with delayed meal timing altering the temporal patterns in leptin levels (11, 12). These effects have been partially supported by studies of occupational shift workers. Working the nightshift has been associated with increased leptin levels, a higher prevalence of metabolic syndrome, impaired glucose tolerance, and insulin resistance (16–21). Numerous studies have reported associations between shiftwork and a higher prevalence of cardiovascular and metabolic disease (22).

Therefore, it is thought that circadian misalignment disrupts key metabolic rhythms, which in turn increases the risk of cardiometabolic disease among nightshift workers. However, it is possible that acute studies of circadian misalignment may not adequately represent the effects of long-term circadian misalignment stemming from working the nightshift. Only a handful of studies have examined metabolic hormones such as insulin and leptin in chronic shift workers, and of these, all but one study measured insulin and/or leptin at only one or two time points over a 24-h period (16–18, 20, 21) and one study conducted repeated measurements of glucose and insulin over a 24-h period (19). To our knowledge, there are no studies that have examined both insulin and leptin hourly over a 24-h period among day versus night shift workers.

Therefore, we investigated whether the 24-h rhythms in insulin and leptin levels differ between chronic dayshift and nightshift workers. To do so, we conducted a secondary data analysis, leveraging data from an existing study that investigated whether cardiometabolic risk factors, central clock hormonal rhythms, and peripheral clock transcriptional rhythms varied in dayshift versus nightshift nurses (23, 24). We hypothesized that nightshift nurses would exhibit higher mean insulin levels but lower mean leptin levels compared to dayshift nurses. Additionally, we conducted a *post hoc* exploratory analysis in the subgroup of nightshift nurses to examine whether the timing of food intake modulates these effects.

## Materials and Methods

### Participants

**Figure 1:**
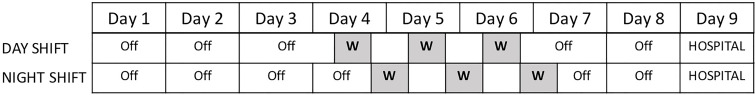
Nine-Day Testing Protocol. Nurses were scheduled to work three consecutive 12-h shifts (greyed out). Nurses tracked their food intake during their work period starting at the beginning of their first work shift through the end of their last work shift. Nurses were admitted for a 24-h hospital stay beginning on the morning of day 9.

A convenience sample of female dayshift (07:00 – 19:00; n = 8) and nightshift (19:00 – 07:00; n = 10) nurses aged 25-61 years were recruited *via* flyers posted at the University of Alabama at Birmingham (UAB) Hospital. Full details on the participants and study protocol are reported elsewhere (23, 24). Briefly, nurses were eligible if they 1) were employed full time (>26 hours per week; 2) worked three consecutive 12-h shifts followed by three or four consecutive days off for at least three weeks prior to study enrollment (see **Figure 1** for shift schedule); and 3) had at least one year of work experience on their current shift (i.e., dayshift or nightshift). Nurses were excluded from the current study if they 1) had a psychiatric disorder as assessed by the Mini-International Neuropsychiatric Interview (MINI); 2) worked multiple jobs; 3) reported current cigarette smoking, excessive alcohol use (more than 15 units per week), or illegal drug use; 4) reported using potentially sedating medications in the 30 days prior to enrollment; or 5) reported using medications to treat hypertension, diabetes, obesity, or hyperlipidemia. The study was approved July 2014 by the UAB Institutional Review Board (F120305016), and all participants provided written informed consent prior to study participation. Nurses were compensated $150 for the inpatient study.

### Diurnal Pattern of Food Intake

At baseline, participants self-reported their daily food intake (type, quantity, and timing for each eating event) using a custom-designed form for 8 consecutive days starting at the beginning of their shift (i.e., for nightshift nurses, between 19:00 on the first workday and 07:00 on the final workday). Energy intake for each food or beverage item was calculated using Nutrition Data System for Research (NDSR), a computer-based software application developed at the University of Minnesota Nutrition Coordinating Center (NCC) (25, 26). To analyze the distribution of energy intake across the day, energy intake was binned into 3-h intervals coinciding with the timing of blood draws, as described below. Nightshift nurses were considered to eat predominantly during the nighttime if >50% of their food intake occurred between 21:00-06:00 (n = 5) and to eat predominantly during the daytime if >50% of their food intake occurred between 06:00 – 21:00 (n = 4). One nurse was excluded for having a pattern that did not fit into either category.

### Inpatient Testing Protocol

On day 9 of the study, participants were admitted to the UAB Hospital Clinical Research Unit (CRU). Participants then completed a 24-h inpatient stay. During the inpatient stay, participants were served three meals and one snack composed of 25% fat, 50% carbohydrate, and 25% protein [as in Scheer et al. (5)] at 09:00 (breakfast), 12:00 (lunch), 15:00 (snack), and 18:00 (dinner). Participants remained recumbent in a hospital bed with limited motion for the duration of the inpatient testing. To minimize the influence of the light-dark (LD) cycle, room lighting was kept at <5 lux, hallway light was blocked *via* an interior room curtain, and participants utilized a combination of blue-light blocking glasses and screen covers on electronic devices.

### Measurement of Insulin and Leptin

Patients were asked to refrain from eating after 21:00 the night prior to inpatient stay. Blood specimens were collected every 3 h beginning at 09:00, for a total of 8 time points. Blood components were immediately separated from anti-coagulated whole blood (5 ml) with density gradient centrifugation (A7054 Histopaque-1077; Sigma-Aldrich, St. Louis, MO). Isolated plasma was immediately flash-frozen in liquid nitrogen and stored at −80°C until assayed. Plasma leptin and insulin were assayed using Milliplex HADK2MAG-61K (Sigma-Aldrich, St. Louis, MO), which has intra-assay CVs of 3-5% and 11-13%, respectively.

### Statistical Analysis

All analyses were conducted using two-sided tests with α = 0.05 in the software program *R* (version 4.1.0, R Core Team, 2021). Insulin and leptin values were modeled using generalized additive models. For each model, we treated shift timing (dayshift vs. nightshift) as a fixed effect (representing the mean 24-h effect), modeled the main effect of time of day using a penalized spine, included an interaction term between time of day (as a categorical variable) and shift timing, and treated participant number as a random effect. We also conducted a *post hoc* exploratory analysis among the subgroup of nightshift nurses to test whether eating predominantly during the daytime versus nighttime affects insulin or leptin levels, using similar generalized additive models. Values are reported as mean ± SEM, unless otherwise stated.

## Results

### Participants

Nurses were predominately White (87.5% of dayshift nurses and 80.0% of nightshift nurses, respectively) and all were female. Comparison of demographic and metabolic parameters by shift type are presented in **Table 1** [note that data from all dayshift and 9 out of 10 nightshift nurses were previously published (23)]. Dayshift and nightshift nurses did not significantly differ in age, BMI, average 24-hr energy intake, or most metabolic parameters assessed (fasting glucose, fasting triglycerides, total cholesterol, and LDL cholesterol). However, nightshift nurses exhibited significantly higher HDL cholesterol compared to dayshift nurses.

**Table 1:** Metabolic Characteristics of Dayshift Versus Nightshift Nurses. Mean ± SD.

	Dayshift (N = 8)	Nightshift (N = 10)	*p*
Age (years)	35 (9)	30 (1)	0.31
BMI (kg/m^2^)	28.5 (8.4)	27.2 (5.6)	0.70
Fasting glucose (mg/dl)	88 (8)	89 (10)	0.87
Fasting triglycerides (mg/dl)	99 (51)	72 (58)	0.31
Total cholesterol (mg/dl)	171 (42)	170 (34)	0.98
HDL cholesterol (mg/dl)	45 (10)	58 (13)	0.04
LDL cholesterol (mg/dl)	109 (35)	96 (37)	0.43
Total energy intake (kcal/day)	1715 (419)	1707 (409)	0.97

The majority (90%, n = 9) of nightshift nurses slept primarily during the night while off-work, specifically utilizing a sleep strategy type defined in Gamble et al. (27) and Petrov et al. (28) as a Switch Sleeper, which is characterized by switching between nighttime sleeping and daytime sleeping by increasing sleep duration on the first day of nightshift work or the first day off. One nurse exhibited a pattern of frequent daytime napping, which Gamble et al. (27) refer to as a Nap Proxy sleep strategy (≥1 h napping for at least 4 off-work days when they would normally be asleep when working night-shift). Additionally, during the baseline study period, three of the night-shift nurses were called off their third shift and, therefore, worked only two shifts. Importantly, there were no differences between dayshift and nightshift nurses in sleep duration, sleep efficiency, wake after sleep onset, or the fragmentation index on days off, as measured by actigraphy (24).

### Insulin

**Figure 2:**
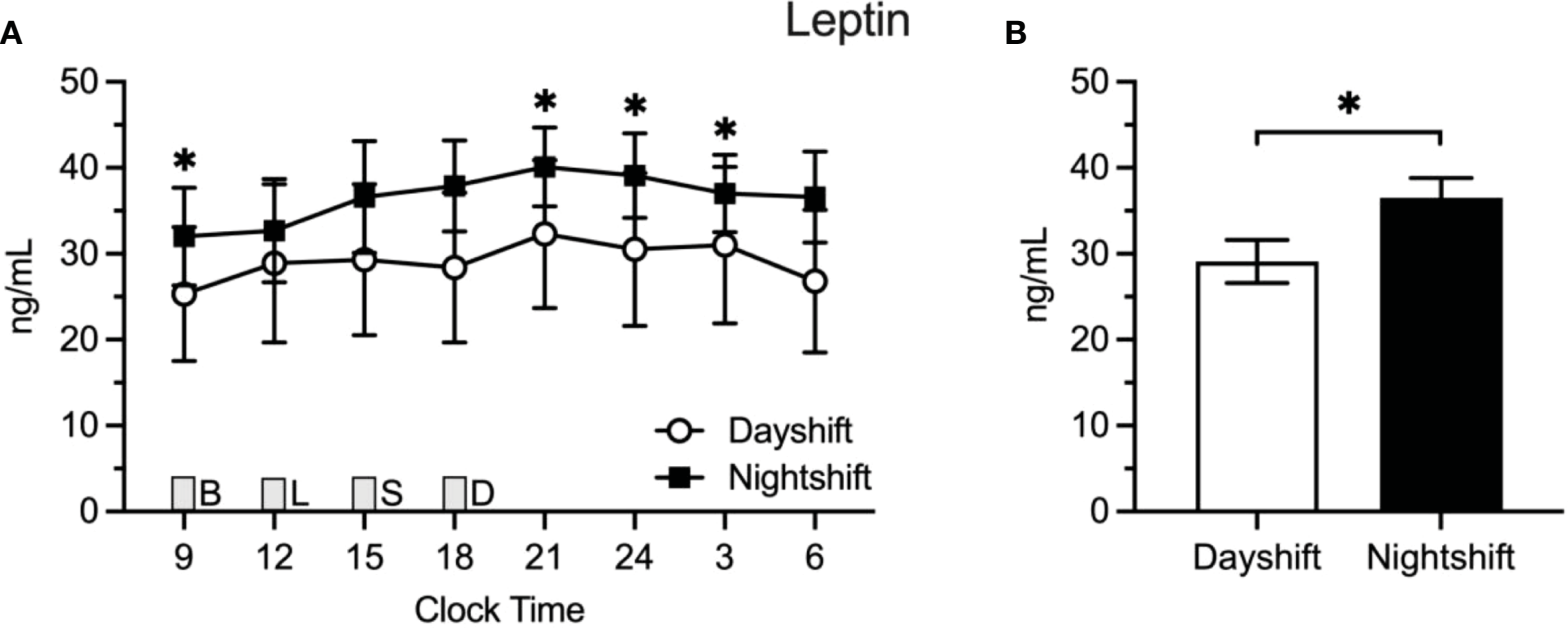
Insulin Levels in Dayshift (*n* = 8) Versus Nightshift Nurses (*n* = 10). **(A)** Raw values ± SEMs for insulin as a function of clock time and shift type. **(B)** Mean 24-h values ± SEM for insulin by shift type, as derived from generalized additive models. Meals were served at 09:00 (B, breakfast), 12:00 (L, lunch), 15:00 (S, snack), and 18:00 (D, dinner) and are designated with a grey box. *p < 0.05.

**Figure 2A** displays the raw means ± SEMs for insulin as a function of clock time among dayshift versus nightshift nurses. Time of day had a similar effect on insulin levels within each group, permitting the use of a single spline to model the effects of time. Using generalized additive modeling, mean values of insulin across the 24-h cycle were 18.9 ± 2.8 mU/L and 30.5 ± 2.5 mU/l among dayshift and nightshift nurses, respectively (**Figure 2B**). Thus, working the nightshift was associated with a 11.6 ± 3.8 mU/l increase in mean insulin levels relative to working the dayshift (p = 0.003). Individual insulin values were higher in nightshift nurses at every time point tested (p ≤ 0.02).

### Leptin

**Figure 3:**
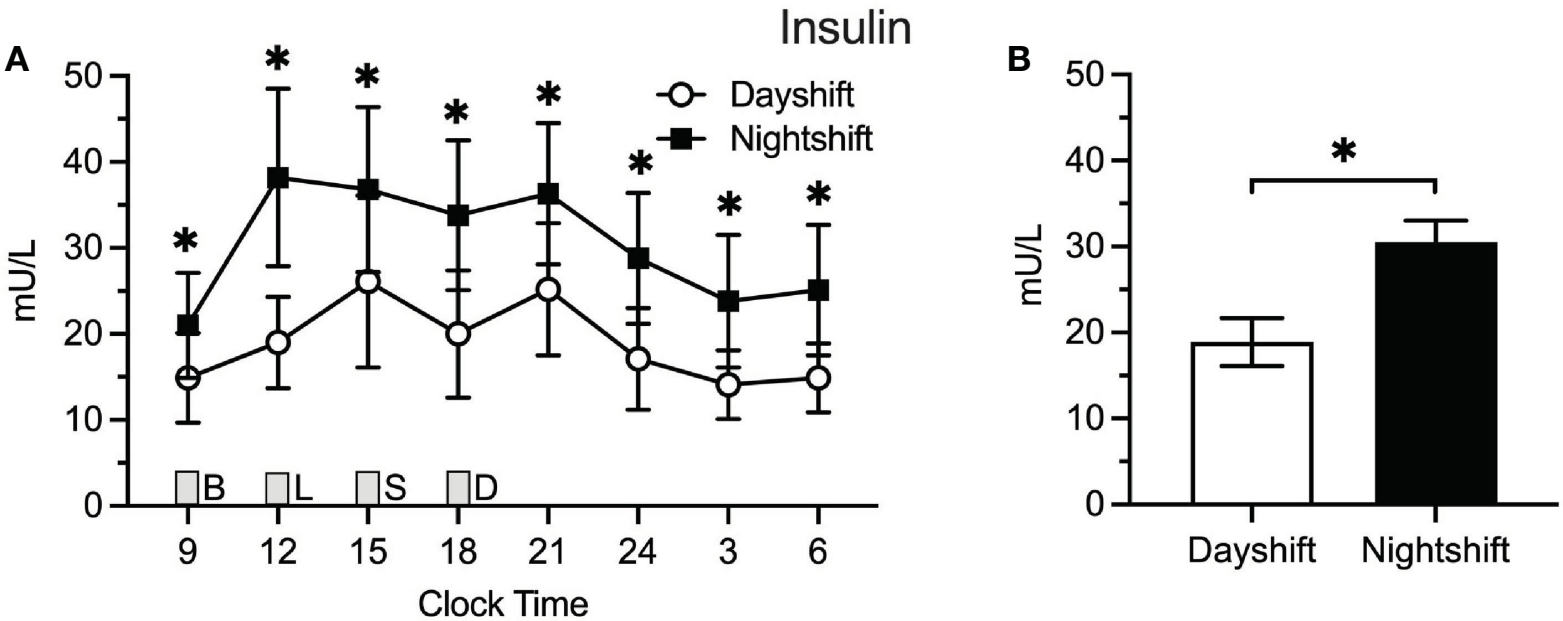
Leptin Levels in Dayshift (*n* = 8) Versus Nightshift Nurses (*n* = 10). **(A)** Raw values ± SEMs for leptin as a function of clock time and shift type. **(B)** Mean 24-h values ± SEM for leptin by shift type, as derived from generalized additive models. Meals were served at 09:00 (B, breakfast), 12:00 (L, lunch), 15:00 (S, snack), and 18:00 (D, dinner) and are designated with a grey box. *p < 0.05.

**Figure 3A** displays the raw means ± SEM for leptin across the 24-h cycle among dayshift versus nightshift nurses. Time of day had a similar effect on leptin levels within each group. Mean leptin levels were 29.1 ± 2.5 ng/ml and 36.5 ± 2.3 ng/ml among dayshift and nightshift nurses, respectively (**Figure 3B**). Thus, working the nightshift was associated with a 7.4 ± 3.4 ng/ml increase in leptin, relative to working the dayshift (p = 0.03). Individual leptin values were higher in nightshift nurses in the morning and at nighttime, whereas they fell short of statistical significance at other times of day.

### Timing of Food Intake Among Nightshift Nurses

**Figure 4:**
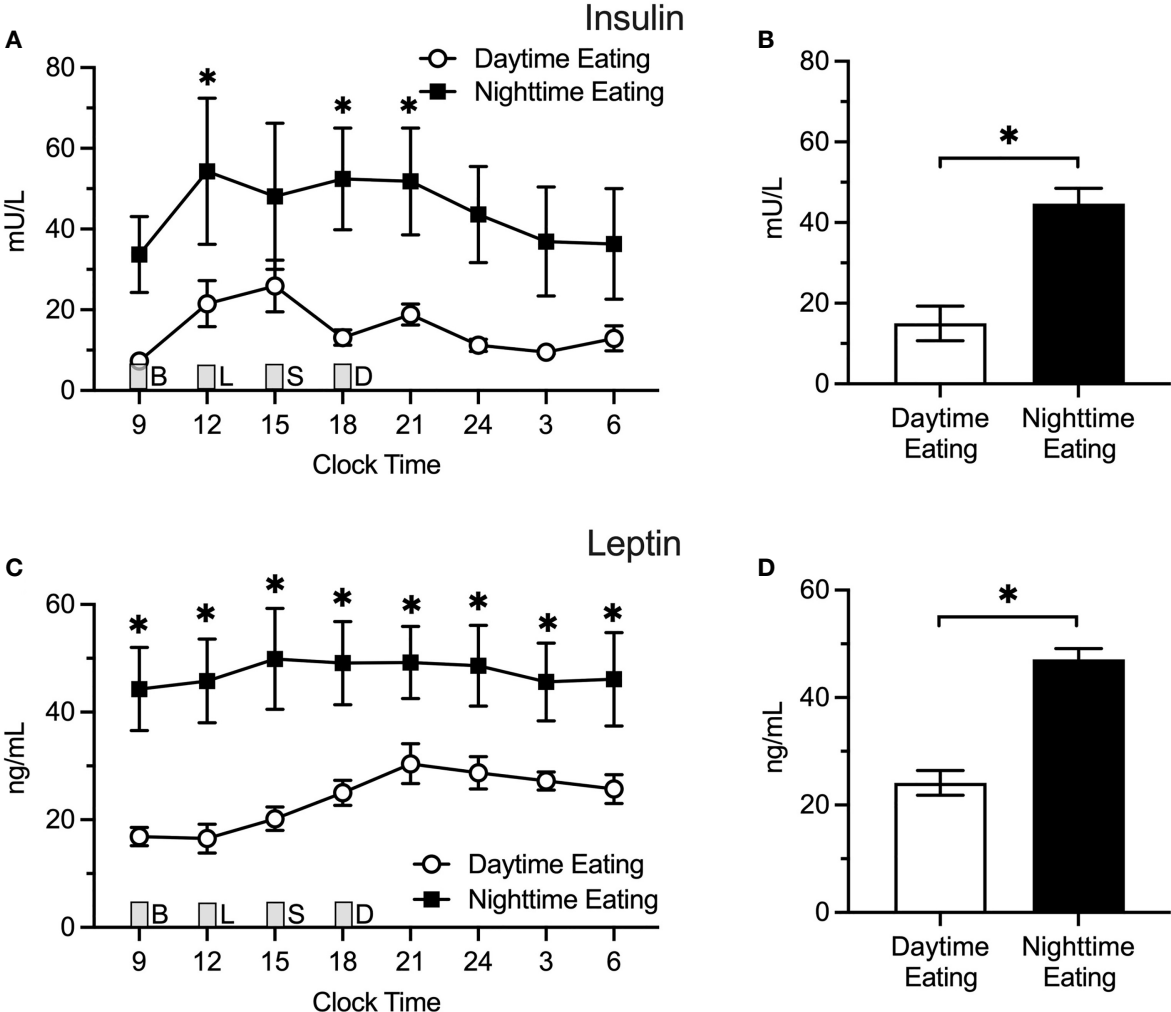
Insulin and Leptin Levels in Predominant Daytime Eating (*n* = 4) Versus Predominant Nighttime Eating (*n* = 5) Nightshift Nurses. Nighttime eating in nightshift nurses was associated with higher insulin and leptin levels. **(A, C)** Raw values ± SEMs for insulin and leptin as a function of clock time and shift type. **(B, D)** Mean 24-h values ± SEM for insulin and leptin by shift type, as derived from generalized additive models. Meals were served at 09:00 (B, breakfast), 12:00 (L, lunch), 15:00 (S, snack), and 18:00 (D, dinner) and are designated with a grey box. *p < 0.05.

We also conducted a *post hoc* analysis among nightshift nurses to test whether predominantly eating during the daytime (n = 4) versus nighttime (n = 5) while actively working the nightshift affects insulin or leptin levels (**Figure 4**). Using generalized additive modeling, eating predominantly at night (21:00 – 06:00) was associated with more than two-fold higher insulin levels relative to eating predominantly during the daytime (44.7 ± 4.3 vs. 15.0 ± 4.3 mU/L; Δ=29.7 ± 5.7 mU/L; p < 0.0001. Individual insulin values were higher at 12:00, 18:00, and 21:00 h in nurses who ate predominantly at nighttime. Similarly for leptin, eating predominantly at nighttime was associated with a nearly two-fold increase in leptin levels (47.1 ± 2.0 vs. 24.1 ± 2.3 ng/ml; Δ = 23.1 ± 3.1 ng/ml; p < 0.0001) relative to eating predominantly during the daytime. Individual leptin values were higher in nurses who ate at nighttime for all time points tested (p ≤ 0.04). Interestingly, there were no differences in mean insulin (Δ = -3.8 ± 3.3 mU/L; p = 0.25) or leptin (Δ = -6.0 ± 4.4 ng/mL; p = 0.18) levels between dayshift nurses and nightshift nurses who ate predominantly during the daytime.

## Discussion

While several studies have examined the impact of circadian misalignment on metabolic hormones using simulated models of shiftwork, few studies have characterized these associations in chronic shift workers. One study examined glucose and insulin levels over a 24-h period among chronic shift workers but did not examine leptin (19). Therefore, we examined 24-h rhythms in insulin and leptin among dayshift and nightshift hospital nurses to determine how nightshift work may affect the rhythms of these key hormones. Compared with dayshift nurses, nightshift nurses exhibited significantly higher insulin and leptin levels across the 24-h day. The magnitude of these differences were moderate and clinically meaningful, as working the nightshift was associated with 11.6 ± 3.8 mU/L and 7.4 ± 3.4 ng/ml higher insulin and leptin levels, respectively. We found that working the nightshift appeared to influence only the mean values and did not induce any changes in the temporal patterns of circulating insulin and leptin.

Our results for insulin are corroborated by studies employing simulated shift work or circadian misalignment protocols, which have reported that circadian misalignment increases insulin levels (5, 13, 14, 29). For example, Scheer et al. (5) showed that a 12-h phase delay in sleep, activity, and food intake increased 24-h mean insulin levels by 22% compared to no phase delay. Additionally, a large population-based study of 1,351 workers found that rotating shift workers exhibited higher fasting insulin values compared to their dayshift counterparts, which contributed to a 1.5-fold greater risk of metabolic syndrome when combined with other observed risk factors (30). Our study corroborates these findings but adds that chronically performing nightshift work raises insulin levels, even on non-workdays. Our finding of higher insulin levels likely reflects an underlying increase in insulin resistance, as both acute circadian misalignment and chronic nightshift work induce insulin resistance (13, 14, 19–21, 31–35). Mechanistically, since circadian misalignment alters the melatonin rhythm, which in turn affects beta-cell function and insulin resistance, it is possible that nightshift work and/or nighttime eating may mediate effects on insulin levels *via* melatonin. However, in our small study, we found no correlations between the amplitude or phase of melatonin and mean 24-hour insulin or leptin values (data not shown). Further research is therefore needed on the role of central and peripheral clocks in potentially contributing to the elevation in insulin levels we observed in nightshift nurses.

However, our finding of higher leptin levels is largely contrary to prior literature. The few studies that have previously examined the acute effects of circadian misalignment and/or simulated shift work have reported decreases in 24-h mean leptin levels (5, 15, 36). This discrepancy may be due to differences between short-term versus long-term responses to circadian misalignment, given that the aforementioned studies used experimental protocols lasting 6 to 25 days, whereas the shift workers in our study had been working night shifts for at least one year. Consistent with our findings, one small preliminary study conducted among workers of different shift types also reported higher mean leptin levels among early morning and nightshift workers compared to dayshift workers; however, the difference between nightshift versus dayshift workers in the prior study was only statistically significant at one time point (12:00) (37). We found a statistically significant elevation in leptin levels at noon among nightshift workers who ate at nighttime, but not in the full sample of nightshift nurses. Since previous studies used different testing protocols, it is unclear whether the differences we observed were due to the study protocol or due to other factors. Nonetheless, leptin levels were higher when aggregated across the day in nightshift nurses. Since leptin regulates satiety and food intake and working the nightshift is associated with obesity, one possible explanation for our findings is that the higher levels of leptin reflect an underlying state of leptin resistance. We speculate that chronic circadian misalignment may induce leptin resistance—a state characterized by reduced satiety, over-consumption of nutrients, and increased total body mass—and, in turn, adipose tissue attempts to compensate by producing higher levels of circulating leptin. This hypothesis may provide a mechanistic link through which circadian misalignment promotes weight gain and obesity. However, dayshift and nightshift nurses in our sample of young adults did not differ in terms of BMI. Regardless of the underlying mechanisms, our finding of higher leptin levels among chronic nightshift workers merits further research.

In an exploratory analysis of nightshift nurses, we also found that nurses who consumed most of their calories during the nighttime on workdays exhibited significantly higher 24-h insulin and leptin levels compared to nurses who predominantly ate during the daytime on workdays. The effects were so large that eating predominantly during the nighttime was associated with roughly two-fold (or higher) levels of insulin and leptin. Interestingly, there was no difference between mean insulin and leptin levels between dayshift nurses and nightshift nurses who ate predominantly during the daytime on workdays, suggesting that eating predominantly at night was driving the increases in insulin and leptin, not the nightshift work itself. These finding are consistent with another recent study using an acute circadian misalignment protocol, which found that eating during the daytime during simulated nightshift work may prevent internal circadian misalignment and glucose intolerance (38). Our findings are also consistent with both laboratory and field studies establishing a connection between nighttime food intake and metabolic outcomes (38–40). However, since our sample size was small, our findings should be viewed with caution and interpreted as hypothesis-generating, until it is replicated. Of note, while a sub-sample of nightshift nurses ate predominately at night when working, all nightshift nurses ate predominately during the day when not working (23). Future research is needed to determine the metabolic consequences of intraindividual variability in meal timing resulting from shiftwork (i.e., switching timing of food intake during work versus off-work days).

Our study has both strengths and limitations. Our study is the first to examine the associations of chronic nightshift work with alterations in 24-h rhythms in insulin and leptin in an occupational shiftwork population. We also collected detailed information on the time of day of food intake across several consecutive days, which enabled us to examine the interaction between working the nightshift and meal timing. However, our results should be interpreted in the context of several limitations. The primary limitation of this study is its small sample size and our recruitment of a convenience sample from only a single hospital system, which may introduce sampling bias. For instance, it is possible that the patterns of predominant nighttime eating observed among nightshift workers in our study may not be representative of the broader population of nightshift nurses or other occupational shiftwork groups. We only analyzed data on the pattern of food intake on workdays. Therefore, further examination of differences in the timing of food intake on workdays versus non-workdays will be an important goal for future studies. Other limitations include the self-reported nature of the nurses’ dietary food intake, as well as the cross-sectional observational design, which necessarily precludes determination of causality and/or directionality of the examined relationships. Lastly, our sample consisted primarily of younger white females, and the effects may be moderated by age (including menopause status), sex, and/or race/ethnicity.

This study provides novel findings in an occupational population suggesting that nightshift work and/or eating predominantly at night may be risk factors for elevated insulin and leptin levels. These alterations in metabolic rhythms may also explain how nightshift work leads to insulin resistance and obesity. Conversely, although nightshift work may raise insulin and leptin levels, eating during the daytime on workdays may attenuate some of the negative effects of nightshift work on metabolic pathways. More rigorous studies are needed among a larger, more diverse sample to clarify the complex relationships present among chronic shift work, meal timing on workdays compared with non-workdays, and metabolic health. Nevertheless, this study serves as a foundation for future research and motivates future efforts toward establishing empirically-based, circadian-informed dietary guidelines for shift workers to curb the excess burden of cardiometabolic disease among this population.

## Data Availability Statement

The raw data supporting the conclusions of this article will be made available by the authors, without undue reservation.

## Ethics Statement

The studies involving human participants were reviewed and approved by University of Alabama at Birmingham Institutional Review Board. The patients/participants provided their written informed consent to participate in this study.

## Author Contributions

KG recruited participants, designed the study, contributed to data collection, processed data, obtained funding, and reviewed/edited the manuscript. HM analyzed food logs, processed data, and contributed to data collection. RJ contributed to data collection, analyzed samples and processed data. CP performed statistical analyses, drafted sections of the manuscript, and reviewed literature. GG processed data and reviewed/edited the manuscript. HM and ST processed data, assisted with statistical analyses, researched literature, and wrote the manuscript. All authors contributed to the article and approved the submitted version.

## Funding

This research was supported by the UAB Center for Clinical and Translational Science (CCTS) Grant Number ULITR000165, the UAB Department of Vision Sciences, the UAB Comprehensive Diabetes Center, the UAB Department of Psychiatry Office of Clinical Research, and American Heart Association Grant Number 19CDA34660139 (ST).

## Conflict of Interest

The authors declare that the research was conducted in the absence of any commercial or financial relationships that could be construed as a potential conflict of interest.

## Publisher’s Note

All claims expressed in this article are solely those of the authors and do not necessarily represent those of their affiliated organizations, or those of the publisher, the editors and the reviewers. Any product that may be evaluated in this article, or claim that may be made by its manufacturer, is not guaranteed or endorsed by the publisher.
