# Erratum: Nightshift work and nighttime eating are associated with higher insulin and leptin levels in hospital nurses

**DOI:** 10.3389/fendo.2023.1240127

**Published:** 2023-06-23

**Authors:** Frontiers Production Office

**Affiliations:** Frontiers Media SA, Lausanne, Switzerland

**Keywords:** circadian misalignment, meal timing, insulin, Leptin, shiftwork

Due to a production error, there was a mistake in **Figure 2** as published. It was a duplicate of Figure 3. The correct **Figure 2** appears below.

**Figure 2:**
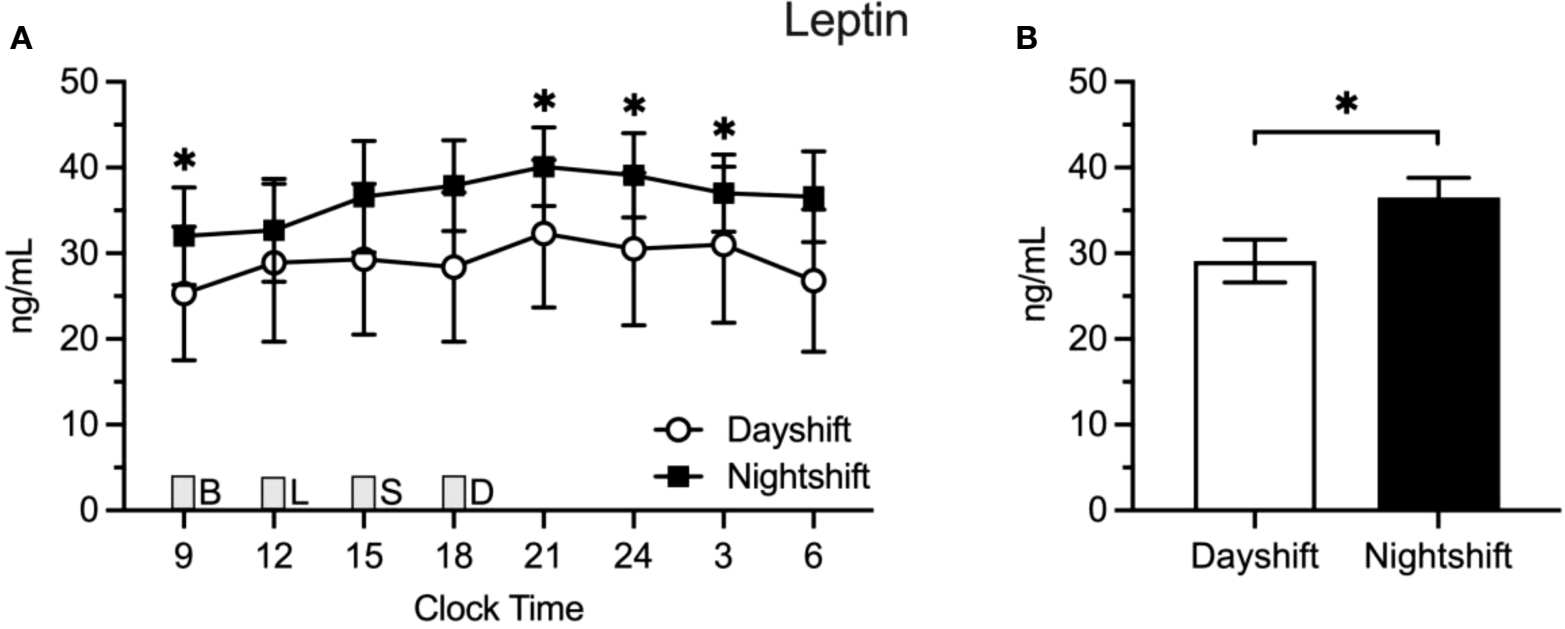
Insulin Levels in Dayshift (*n* = 8) Versus Nightshift Nurses (*n* = 10). **(A)** Raw values ± SEMs for insulin as a function of clock time and shift type. **(B)** Mean 24-h values ± SEM for insulin by shift type, as derived from generalized additive models. Meals were served at 09:00 (B, breakfast), 12:00 (L, lunch), 15:00 (S, snack), and 18:00 (D, dinner) and are designated with a grey box. *p < 0.05.

The publisher apologizes for this error. The original article has been updated.

