# Erratum: Nightshift work and nighttime eating are associated with higher insulin and leptin levels in hospital nurses

**DOI:** 10.3389/fendo.2024.1528687

**Published:** 2024-11-27

**Authors:** Frontiers Production Office

**Affiliations:** Frontiers Media SA, Lausanne, Switzerland

**Keywords:** circadian misalignment, meal timing, insulin, Leptin, shiftwork

Due to a production error, the captions of **Figures 2** and **3** were swapped. The correct captions appear below.

**Figure 2**

Leptin Levels in Dayshift (n = 8) Versus Nightshift Nurses (n = 10). **(A)** Raw values ± SEMs for leptin as a function of clock time and shift type. **(B)** Mean 24-h values ± SEM for leptin by shift type, as derived from generalized additive models. Meals were served at 09:00 (B, breakfast), 12:00 (L, lunch), 15:00 (S, snack), and 18:00 (D, dinner) and are indicated by a gray box. *p < 0.05.

**Figure 3**

Insulin Levels in Dayshift (n = 8) Versus Nightshift Nurses (n = 10). **(A)** Raw values ± SEMs for insulin as a function of clock time and shift type. **(B)** Mean 24-h values ± SEM for insulin by shift type, as derived from generalized additive models. Meals were served at 09:00 (B, breakfast), 12:00 (L, lunch), 15:00 (S, snack), and 18:00 (D, dinner) and are indicated by a gray box. *p < 0.05.

The publisher apologizes for this mistake. The original version of this article has been updated.

